# Atlas-level single-cell integration and clustering-free differential expression analysis with GEDI 2.0

**DOI:** 10.1093/bioinformatics/btag334

**Published:** 2026-05-22

**Authors:** Arsham Mikaeili Namini, Ali Saberi, Hamed S Najafabadi

**Affiliations:** Department of Human Genetics, McGill University, Montreal, QC H3A 1Y2, Canada; Victor P. Dahdaleh Institute of Genomic Medicine, McGill University, Montreal, QC H3A 0G1, Canada; Victor P. Dahdaleh Institute of Genomic Medicine, McGill University, Montreal, QC H3A 0G1, Canada; Department of Electrical and Computer Engineering, McGill University, Montreal, QC H3A 0E9, Canada; Department of Human Genetics, McGill University, Montreal, QC H3A 1Y2, Canada; Victor P. Dahdaleh Institute of Genomic Medicine, McGill University, Montreal, QC H3A 0G1, Canada; McGill Centre for RNA Sciences, McGill University, Montreal, QC H3G 0B1, Canada

## Abstract

**Motivation:**

GEDI is a generative framework for multi-sample, multi-condition single-cell analysis that performs batch correction, latent representation learning, and clustering-free differential expression within a unified model. However, the original implementation suffered from prohibitive memory use and runtime, preventing its application to modern atlas-scale datasets.

**Results:**

We present GEDI 2.0, a complete high-performance reimplementation featuring a standalone C++ computational core with pre-allocated workspaces, strict sparse-matrix preservation, optimized BLAS routines, and multi-threaded block-coordinate descent. Across extensive benchmarks spanning up to 500 000 cells and 10 000 features, GEDI 2.0 achieves 40%–63.6% mean reduction in peak memory, 2.98× mean single-threaded speedups, and up to 11.5× acceleration with parallel execution, while maintaining full numerical equivalence to the original method. These improvements enable GEDI 2.0 to analyze million-cell datasets, a scale not achievable with the legacy implementation. GEDI 2.0 provides R and Python interfaces and seamless interoperability with common single-cell workflows.

**Availability and implementation:**

Source code, documentation, reproducible codebase, and tutorials are available at https://github.com/csglab/gedi2.

## 1 Introduction

Single-cell RNA sequencing has revealed unprecedented cellular heterogeneity across tissues, individuals, and conditions. However, extracting biological insights from multi-sample, multi-condition datasets remains challenging, as standard workflows treat data integration, differential expression analysis, and pathway inference as separate sequential steps, ignoring their fundamental interdependencies. The GEDI (Gene Expression Decomposition and Integration) framework was recently introduced to address this limitation through a unified generative model (Madrigal *et al.* 2024). By modeling each sample’s gene expression manifold as a transformation of a shared reference space, GEDI enables state-of-the-art cross-sample cell state mapping and harmonization. In addition, by modeling these sample-specific transformations as functions of sample-level variables (such as disease status), GEDI enables cluster-free differential expression analysis, revealing the transcriptomic changes that accompany different sample variables along the continuum of cell states. Also, by modeling the reference expression manifold as a function of gene-level variables (such as pathway belonging, or regulatory connections), GEDI can project pathway and regulatory network activities onto single cells. Finally, GEDI extends these concepts to previously unexplored modalities requiring joint consideration of dual measurements, such as alternative splicing analysis from exon inclusion/exclusion reads.

Despite its theoretical advances, the original GEDI implementation faced a critical limitation: memory consumption scaled prohibitively with dataset size, making analysis of modern single-cell atlases, which often comprise millions of cells from hundreds of samples, impractical or even impossible on standard computational infrastructure. This gap between GEDI’s novel abilities and its computational feasibility motivated us to completely re-engineer its implementation. Here, we present GEDI 2.0, a high-performance reimplementation that eliminates memory redundancy and delivers several-fold speed gains through a C++ computational backend while preserving full numerical equivalence to the original method. These improvements transform GEDI into a modern tool capable of analyzing atlas-level single-cell datasets on standard workstations, democratizing access to its modeling framework for the broader single-cell community.

## 2 Implementation

The core of GEDI can be categorized into three main steps: model setup, latent variable initialization, and iterative block coordinate descent optimization (see the original publication for detailed explanation of the mathematical aspects underlying these steps). The original GEDI implementation had several bottlenecks in each step, including unnecessary sparse-to-dense matrix conversions, repeated data transfers between R wrapper functions and the C++ back-end, and memory duplication where both R and C++ environments maintained separate copies of all parameters. This duplication resulted in memory spikes when the back-end was updated while the R environment retained previous parameter versions. Additionally, within each optimization iteration, parameter updates for individual samples are mathematically independent and can be computed simultaneously, yet the original implementation did not exploit this parallelization opportunity.

To address these issues, we reimplemented GEDI with a pure C++ standalone back-end. The new implementation utilizes pre-allocated C++ workspaces, allocating memory for all model parameters at initialization and performing in-place updates after each iteration. This approach maintains low peak memory usage throughout the optimization process. Sparse matrices are preserved throughout all computations, avoiding unnecessary memory overhead from dense conversions. Multi-threading support has been integrated to exploit the independence of sample-specific parameter updates, allowing performance to scale with available CPU cores. All low-level linear algebra computations were re-engineered using optimized BLAS routines for improved numerical efficiency.

To ensure consistency with the previous version of GEDI, we designed validation tests comparing both implementations on identical datasets with matched parameters and iteration counts. For numerical equivalence testing, we examined all model parameters, including the mean-centered and offset-adjusted latent factor representations and sample-specific factor representations. To isolate potential sources of numerical differences, we conducted controlled experiments replacing specific numerical subroutines (such as QR decomposition) to determine whether any discrepancies arose from algorithmic changes or merely from different numerical library implementations. For performance benchmarking, we evaluated both single-threaded and multi-threaded execution across datasets ranging from 50 000 to 500 000 cells, measuring both peak memory consumption and runtime with varying numbers of CPU threads. The reimplementation as a standalone C++ core maintains full backward compatibility with models trained using the original GEDI and ensures consistent numerical behavior across different computing environments. This core library has been packaged with interfaces for both R and Python, making GEDI 2.0 accessible to the broader computational biology community while maintaining consistent performance across programming environments.

## 3 Results

### 3.1 GEDI 2.0 overview

**Figure 1:**
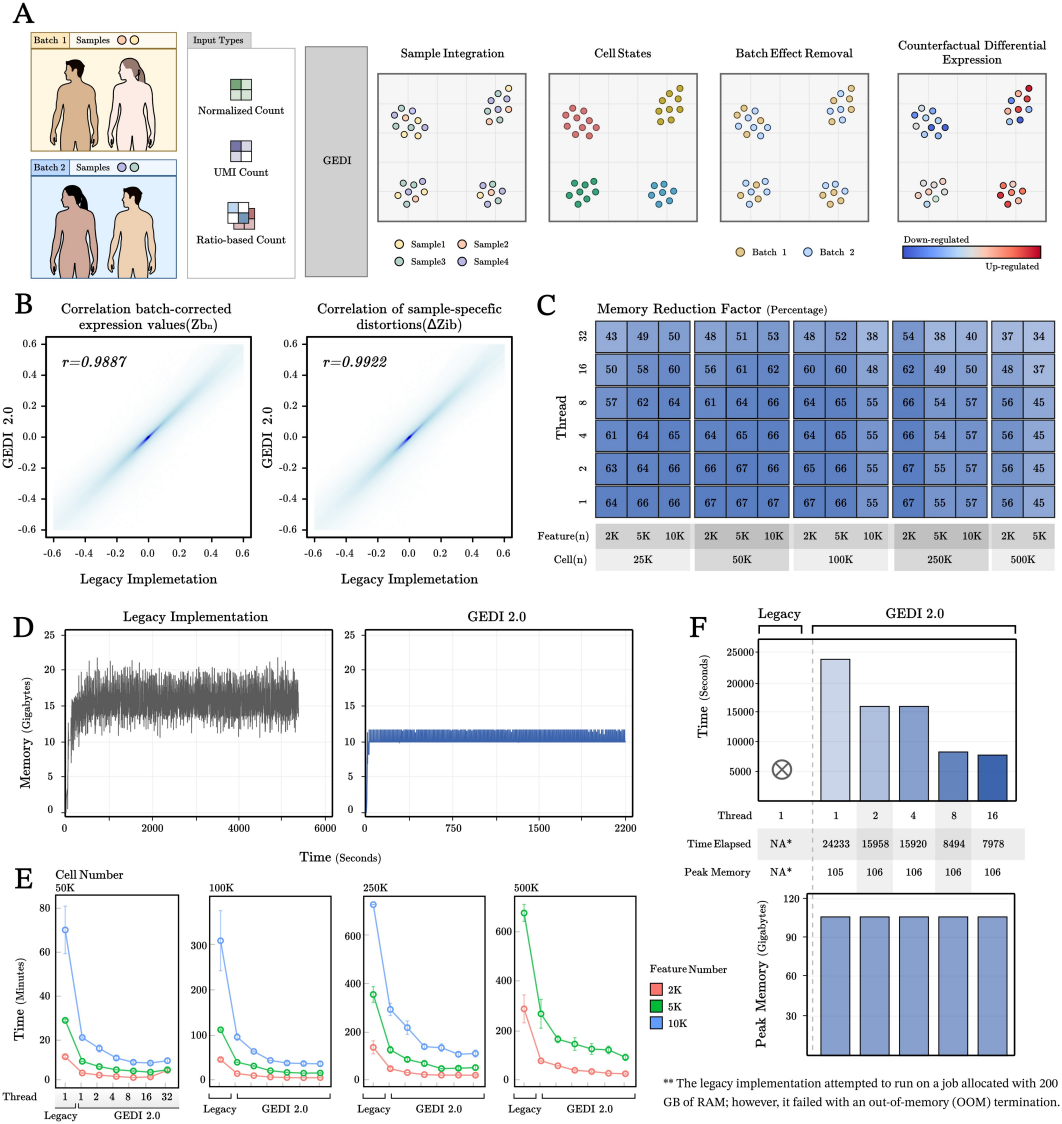
GEDI 2.0 overview and performance. (A) Overview of GEDI 2.0 abilities, inherited from legacy GEDI (Madrigal *et al*. 2024). (B) Comparison of legacy GEDI and GEDI 2.0 with respect to batch-corrected values in the expression space ($Zb_{n}$, left) as well as sample-specific expression distortion ($\Delta Z_{i}b_{n}$, right). (C) Peak memory reduction percentage across a grid benchmark. The heatmap displays the percentage of memory saved by GEDI 2.0 compared to an equivalent Legacy GEDI run. The grid is organized by cell counts (25K–500K) and feature counts (2K, 5K, and 10K) across the columns, while rows represent the number of threads utilized by GEDI 2.0. (D) Memory usage fluctuations for the 50K-cell dataset. (E) Compute time for the grid benchmark. Parallelization efficiency is shown across four datasets of increasing size (50K, 100K, 250K, and 500K cells). Colors represent the number of features (2K, 5K, or 10K, except for the 500K-cell dataset, on which we did not benchmark with 10K features due to legacy GEDI’s memory limitations). The x-axis shows the single-threaded Legacy implementation and GEDI 2.0 scaled from 1 to 32 threads, with the y-axis indicating total compute time in minutes. (F) Compute time (top) and peak memory usage (bottom) for a 1M-cell dataset based on the top 2000 most variable genes. All benchmarking experiments were performed on the Nibi cluster from Digital Research Alliance of Canada using Intel 6972P CPUs.

GEDI 2.0 maintains the full analytical capabilities of the original framework (Fig. 1A) while introducing substantial performance improvements. Specifically, it simultaneously performs batch effect removal, latent space embedding, and clustering-free differential expression analysis. This is achieved by decomposing variation into shared biological structure (reference axes $Z=[z_{1},z_{2},\ldots ,z_{K}]$, where *K* is the number of latent factors) and sample-specific distortions of these axes ($\Delta Z_{i}$, where *i* is the sample index). Each cell *n* is modeled as a point in the shared latent space *Z* with coordinates $b_{n}$. The observed data for each cell is in turn modeled through a data generating distribution whose parameters depend on (Z+ΔZi)$b_{n}$, in addition to gene-specific and cell-specific offsets (size factors), with $Zb_{n}$ providing batch-corrected values in the expression space, and $\Delta Z_{i}b_{n}$ providing the expression distortion in sample *i*. *Z* and ΔZi can optionally be modeled as functions of gene-level and sample-level prior knowledge, as described in more detail in Madrigal *et al.* (2024). This framework enables batch correction while preserving biological heterogeneity, can compute continuous transcriptomic vector fields showing how gene expression shifts along the cell state manifold as sample conditions change (eliminating the need for discrete clustering prior to differential expression analysis), and can infer pathway and gene regulatory network activities from the observed data at single-cell resolution.

### 3.2 Numerical equivalence validation

We validated that the reimplementation maintains mathematical fidelity to the original formulation by training both versions on multiple datasets spanning neuronal, perturbation, single-batch, and multi-technology integration scenarios. These included a 100 000-cell mouse neuronal dataset from the Allen Brain Cell Atlas (Yao *et al.* 2023) and three additional full datasets from SeuratData, i.e. IFNB, PBMC3K, and PBMCsca (Hao *et al.* 2024), all trained using identical parameters (Fig. 1B). Across all datasets, all key model parameters showed near-perfect concordance, including Pearson correlation of 0.9887 for batch-corrected expression values ($Zb_{n}$) across all cells, and Pearson correlation of 0.9922 for sample-specific distortions ($\Delta Z_{i}b_{n}$). Controlled experiments revealed that minor deviations stem exclusively from numerical precision differences in QR decomposition implementations (Eigen for GEDI 2.0 vs. LAPACK for the legacy GEDI) rather than algorithmic changes. When we substituted LAPACK’s QR into the new implementation, results became identical within machine precision ($<10^{-14}$; Supplementary Figs. 1–2), confirming that GEDI 2.0’s optimization procedure remains mathematically unchanged.

### 3.3 Memory efficiency improvements

To systematically assess scalability, we benchmarked GEDI 2.0 across a grid of five dataset sizes, three feature counts [i.e. different numbers of highly variable genes, selected using Seurat’s variance-stabilizing transformation (Stuart *et al.* 2019)], and three replicate runs per configuration, using Allen Brain Cell Atlas. Memory consumption analysis across this benchmark grid revealed 45%–67% (median 66%) reduction in peak usage (Fig. 1C). Moreover, it showed that doubling cells and/or feature size increases peak memory usage sublinearly: about 1.6× in GEDI 2.0 versus 2.3–2.6× in the legacy version (Supplementary Fig. 3A).

Beyond absolute memory reduction, GEDI 2.0 changed memory usage patterns during optimization; e.g. with 50K cells and 5000 features (from Allen Brain dataset), the legacy implementation exhibited continuous fluctuations between 15 and 25 GB, reflecting repeated allocation and deallocation cycles that create memory pressure and risk out-of-memory failures (Fig. 1D-left). In contrast, GEDI 2.0 maintained stable consumption at approximately 11 GB throughout model fitting (Fig. 1D-right), reflecting the pre-allocated workspace strategy that eliminates dynamic memory management overhead.

### 3.4 Computational performance and scalability

Runtime benchmarking demonstrated substantial improvements from both architectural redesign and parallelization (Fig. 1E). Even without parallelism, GEDI 2.0 ran up to ∼3× faster than the legacy implementation, confirming substantial performance gains from improved algorithms and memory access patterns alone. Multi-threading further amplified these benefits, achieving a mean speedup of 6.08× and a maximum of 11.5× for 32-thread execution (Supplementary Fig. 3B).

Performance scaling was near-linear through 8 threads, with diminishing returns beyond 16 threads as synchronization overhead increasingly limits further gains, consistent with the block coordinate descent optimization structure, where sample-specific parameter updates can be perfectly parallelized but global parameter updates require synchronization. Across configurations, 8–16 threads most frequently provided the best balance between runtime and resource usage. These improvements substantially increased practical usability: for a representative 100K-cell configuration averaged across replicates, runtime decreased from near two hours in the legacy implementation to 16 minutes with GEDI 2.0 using moderate parallelism (∼6.8× speedup), with even larger gains observed at 500K-cell scale (∼7.5×), indicating that parallel acceleration becomes more effective for larger datasets.

Lastly, to demonstrate GEDI 2.0’s scalability to atlas-level datasets, we benchmarked performance by increasing the cell count in the mouse neuronal dataset (Yao *et al.* 2023) to 1 million cells with 2000 features. The legacy implementation failed with an out-of-memory error even when allocated 200 GB of RAM. In contrast, GEDI 2.0 successfully completed the analysis using approximately 106 GB of memory in roughly 2 hours with 16 threads (Fig. 1F). This memory requirement falls within the entry tier of high-performance computing resources, making atlas-scale GEDI analysis feasible on high-performance computing (HPC) platforms. As an example of other applications enabled by GEDI 2.0’s scalability, we used DoRothEA transcription factor regulons (Garcia-Alonso *et al.* 2019) to compute and project transcription factor activities onto one million cells from the mouse neuronal dataset (Yao *et al.* 2023), recovering canonical lineage-specific programs such as SPI1 in immune cells and SOX10 in oligodendrocyte-lineage cells (Supplementary Fig. 4).

## 4 Conclusions

GEDI 2.0 transforms a conceptually powerful but computationally prohibitive framework into a practical tool for large-scale single-cell analysis. Through comprehensive architectural redesign, which includes eliminating memory redundancy, implementing efficient C++ algorithms, and enabling multi-threaded execution, we achieved up to 6.8× speedup and 46% memory reduction while maintaining full mathematical equivalence with the original formulation. By packaging the standalone C++ core with both R and Python, GEDI 2.0 enables streamlined adoption of this theoretically robust framework—this is further enhanced now by providing auxiliary functions that enable data conversion and transfer between GEDI and widely used software packages such as Seurat (Hao *et al.* 2024). GEDI 2.0, full documentation, and example datasets and notebooks are freely available at https://github.com/csglab/gedi2.

## Supplementary Material

btag334_Supplementary_Data

## Code availability

Source code, documentation, reproducible codebase, and tutorials are available at https://github.com/csglab/gedi2 under MIT license. Benchmarking data and scripts are available at https://github.com/csglab/gedi2_manuscript.
