# Supplementary material for: Atlas-level single-cell integration and clustering-free differential expression analysis with GEDI 2.0
Source: Bioinformatics. 2026 May 22;42(6):btag334. doi: 10.1093/bioinformatics/btag334 (PMC13224958; doi:10.1093/bioinformatics/btag334)
Supplement: btag334_Supplementary_Data [file btag334_supplementary_data.pdf]

Supplementary Figures for

Atlas-level single-cell integration and clustering-free differential expression analysis with GEDI 2.0

Arsham Mikaeili Namini[1,2], Ali Saberi[2,3], Hamed S. Najafabadi[1,2,4,*]

[1] Department of Human Genetics, McGill University, Montreal, QC, Canada
[2] Victor P. Dahdaleh Institute of Genomic Medicine, Montreal, QC, Canada
[3] Department of Electrical and Computer Engineering, McGill University, Montreal, QC, Canada
[4] McGill Centre for RNA Sciences, McGill University, Montreal, Canada

*** Corresponding author:** Hamed Najafabadi, hamed.najafabadi@mcgill.ca

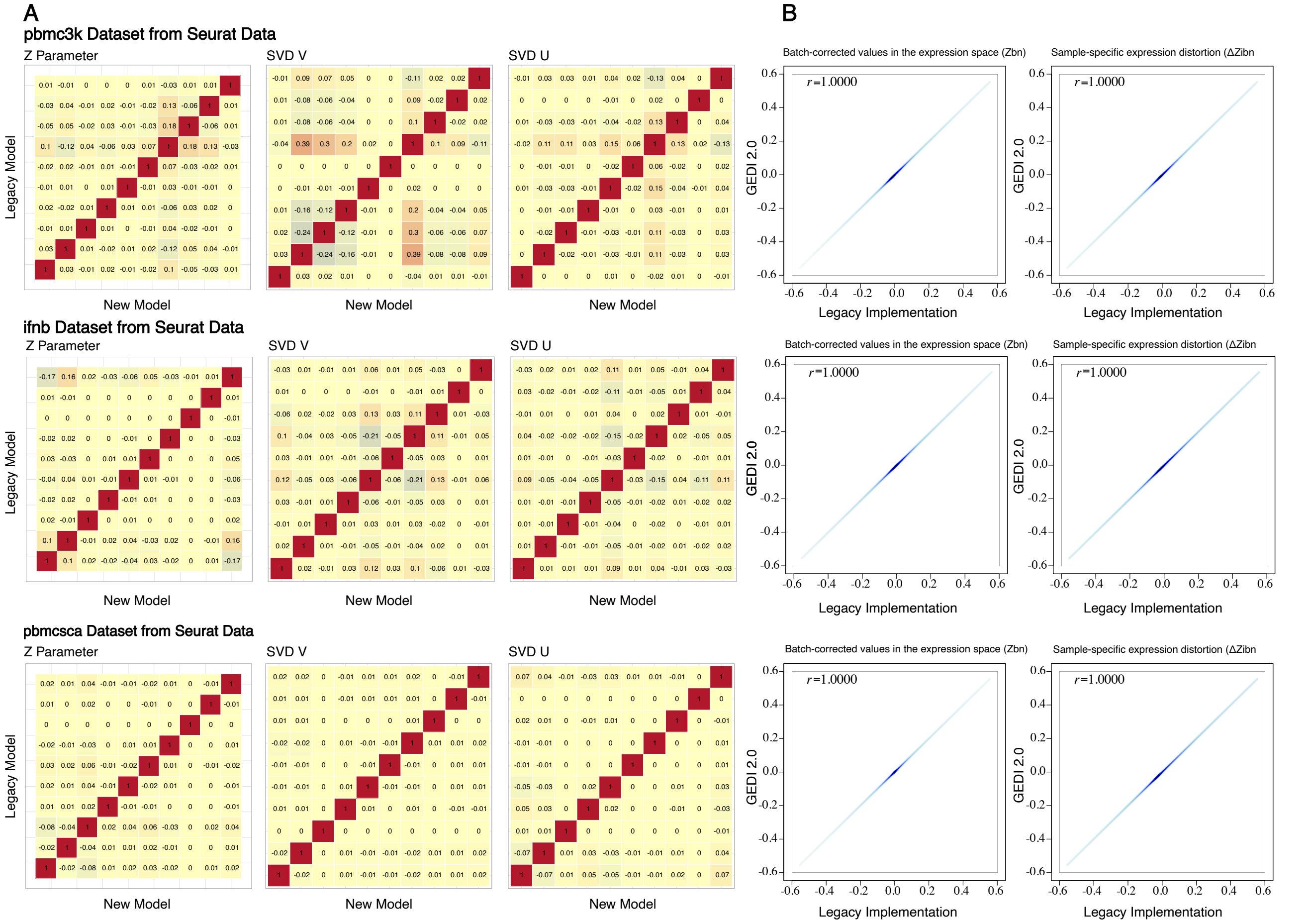

Supplementary Figure 1. Numerical equivalence of GEDI 2.0 and legacy GEDI across three benchmark datasets from (Hao, et al., 2024). (**A**) Each row represents one dataset (pbmc3k, ifnb, pbmcsca), with each heatmap showing pairwise Pearson correlations between parameter sets learned by legacy GEDI and GEDI 2.0 for each latent factor. Within each heatmap, each row corresponds to the parameter vector learned by legacy GEDI, and each column corresponds to the matching parameter vector learned by GEDI 2.0. From left to right, the heatmaps compare: latent factor vectors (***Z*** parameters); right singular vectors (***V***) from the SVD of batch-corrected expression; and left singular vectors (***U***) from the same decomposition. (**B**) The scatter plots compare the resulting model outputs between implementations. The left scatter plot shows batch-corrected expression values in expression space ($\boldsymbol{Zb_n}$), and the right scatter plot shows sample-specific expression distortions ($\Delta \boldsymbol{Z_i b_n}$). Each point represents one value computed by legacy GEDI (x-axis) versus GEDI 2.0 (y-axis).

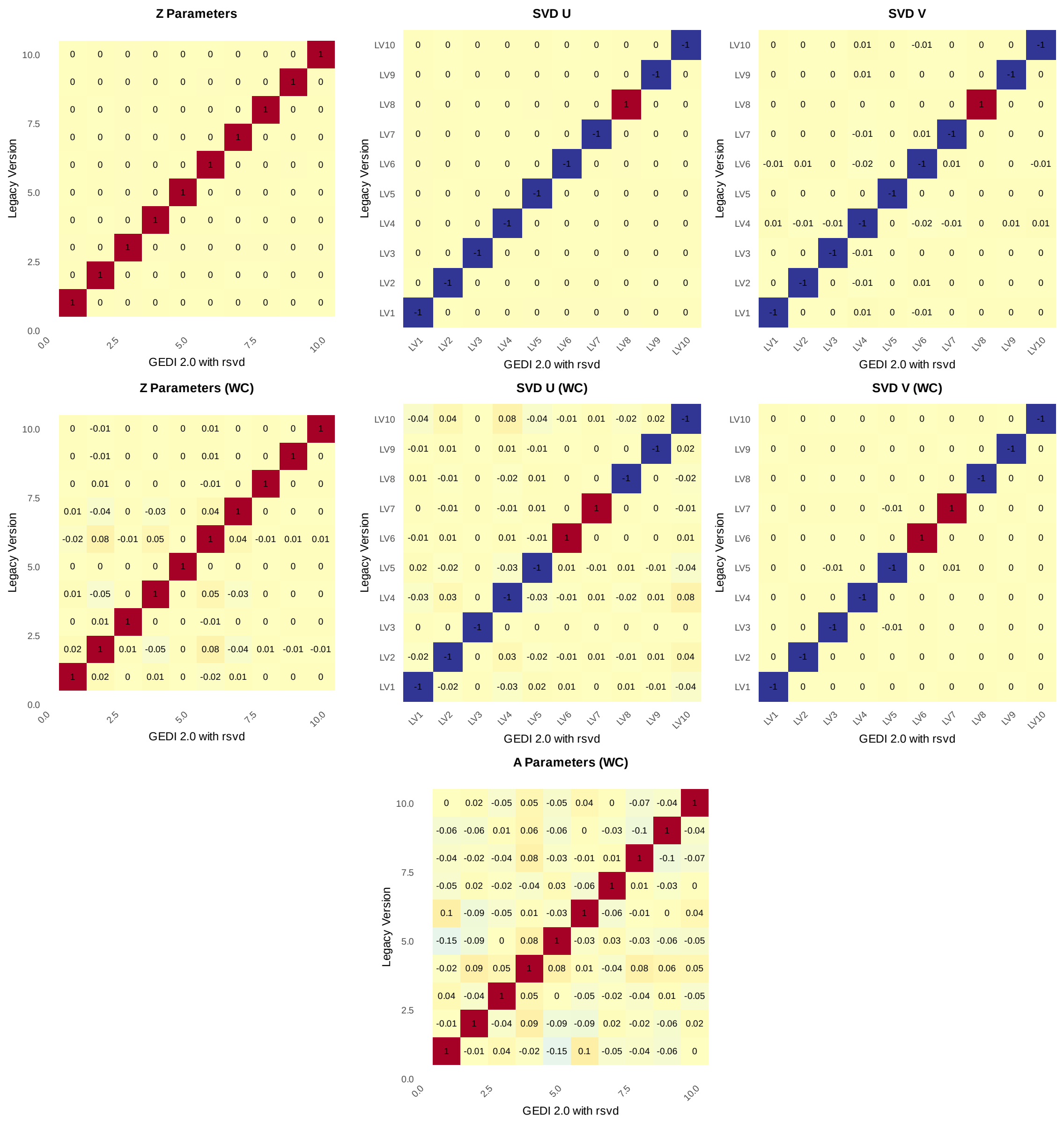

Supplementary Figure 2. Numerical equivalence of GEDI 2.0 and legacy GEDI in the benchmark dataset from (Yao, et al., 2023). Each panel represents the comparison of parameter sets learned by GEDI and GEDI 2.0, fit to the 100K-cell dataset using 10 latent factors. The pairwise Pearson correlations of the parameters learned for each latent factor are shown. For example, in the upper-left panel, each row represents the z vector learned by legacy GEDI for each of the 10 latent factors, and each column represents the z vector learned by GEDI 2.0. In the case of perfect equivalence, a correlation of 1 is expected (or –1, since the sign of the latent factors or their SVD decomposition can be inverted without changing model fit). **Top row:** left: comparison of z vectors (reference axes); middle: comparison of u vectors after SVD decomposition of batch-corrected expression values; right: comparison of v vectors after SVD decomposition of batch-corrected expression values. **Middle row:** same as top, except that the models are fitted using a gene-level prior C matrix. **Bottom:** comparison of a vectors, corresponding to the pathway-latent factor associations learned by GEDI when a gene-level prior C matrix is included.

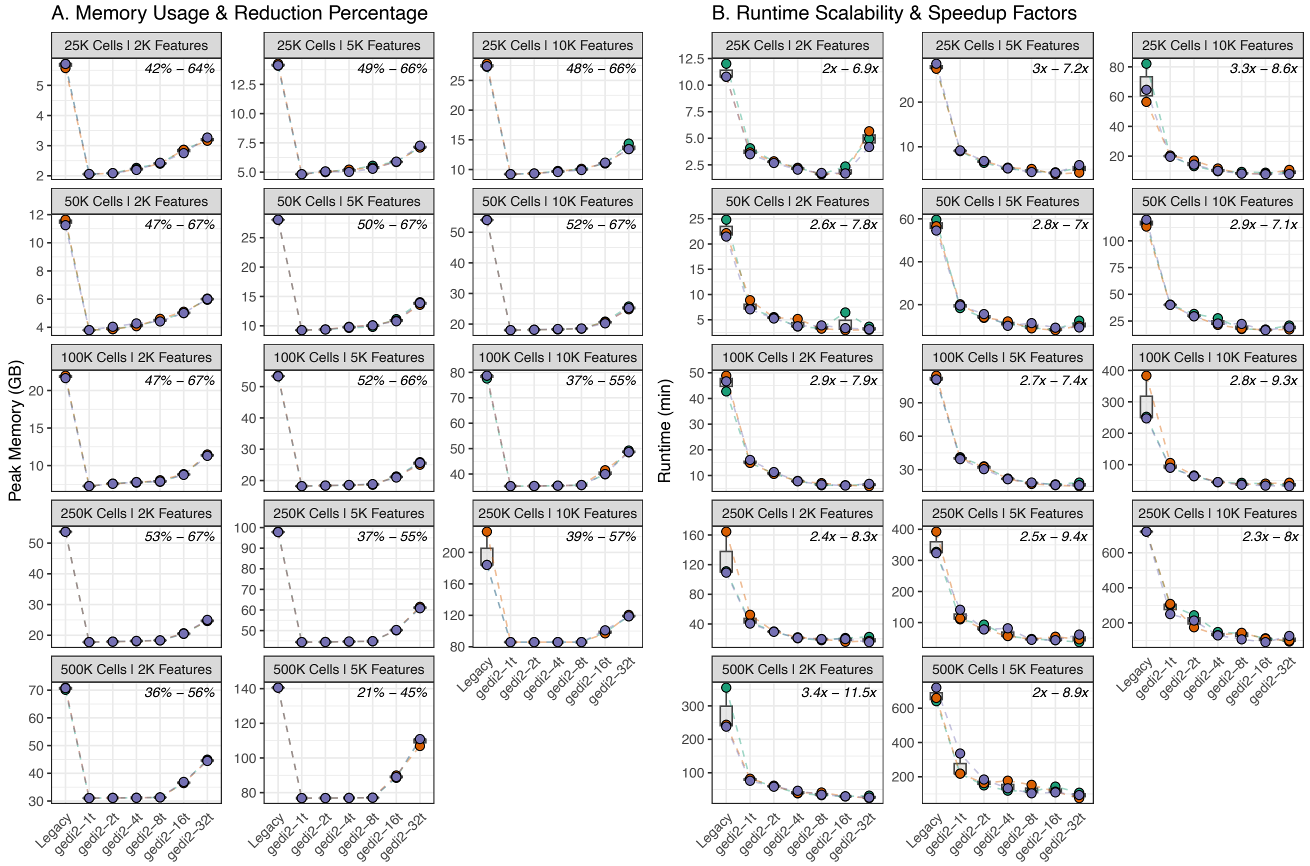

Supplementary Figure 3. Extended scalability benchmarking across dataset sizes, feature counts, and threading configurations. Each panel corresponds to one benchmark configuration defined by a specific number of cells (25K, 50K, 100K, 250K, 500K) and features (2K, 5K, 10K). For each configuration, the legacy GEDI (single-threaded) is compared with GEDI 2.0 executed using 1, 2, 4, 8, 16, and 32 threads. All values are averaged across three independent replicates. (**A**) Peak memory usage (GB). Each point represents the mean peak memory measured during model fitting. Percentages above each panel indicate the range of memory reduction of GEDI 2.0 relative tolegacy GEDI across threading configurations. (**B**) Runtime (minutes). Each point represents the mean runtime required for model fitting. Speedup ranges above each panel indicate the ratio of legacy GEDI runtime to GEDI 2.0 runtime across threading configurations. Benchmark configuration with 500K cells and 10K features could not be evaluated for the legacy implementation due to R vector size limits.

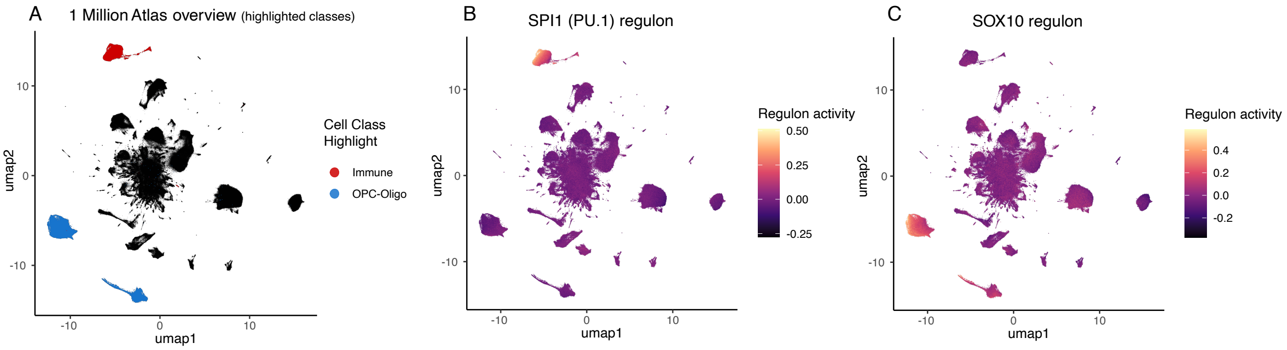

Supplementary Figure 4. Atlas-scale regulon activity highlights lineage-specific transcription-factor programs. (**A**) UMAP embedding of a one-million-cell atlas with immune and OPC-oligodendrocyte classes highlighted. Other cell types are in black. (**B**) SPI1 (PU.1) regulon activity, a canonical regulator of hematopoietic and immune lineages, shows strong enrichment in immune populations. (**C**) SOX10 regulon activity highlights oligodendrocyte lineage cells. Regulon activity was computed by GEDI 2.0 based on DoRothEA (Garcia-Alonso, et al., 2019) regulatory networks.

References

Garcia-Alonso, L., *et al.* Benchmark and integration of resources for the estimation of human transcription factor activities. *Genome Res* 2019;29(8):1363-1375.

Hao, Y., *et al.* Dictionary learning for integrative, multimodal and scalable single-cell analysis. *Nat Biotechnol* 2024;42(2):293-304.

Yao, Z., *et al.* A high-resolution transcriptomic and spatial atlas of cell types in the whole mouse brain. *Nature* 2023;624(7991):317-332.
